# Updated insights on dementia‐related risk of sacubitril/valsartan: A real‐world pharmacovigilance analysis

**DOI:** 10.1111/cns.14195

**Published:** 2023-03-27

**Authors:** Congqin Chen, Lingqing Ding, Fang Fu, Jie Xiao

**Affiliations:** ^1^ Department of Pharmacy, Xiamen Cardiovascular Hospital of Xiamen University, School of Medicine Xiamen University Xiamen China

**Keywords:** Alzheimer's disease, dementia, FAERS, pharmacovigilance study, sacubitril/valsartan

## Abstract

**Aim:**

Sacubitril/valsartan is a new cardiovascular agent characterized by its dual inhibition on the reninangiotensin system (RAS) and the neprilysin. As neprilysin also involved itself in the degradation of amyloid‐β, there is an ongoing concern about the effect of sacubitril/valsartan on cognition, especially in case of long‐term administration.

**Methods:**

The FDA Adverse Event Reporting System (FAERS) was mined between 2015Q3 and 2022Q4 to analyze the association between sacubitril/valsartan and adverse events (AEs) involving dementia. Standardized Medical Dictionary for Regulatory Activities (MedDRA) Queries (SMQs) with “broad” and “narrow” preferred terms (PTs) relevant to dementia was applied to systematically search demented AE reports. The Empirical Bayes Geometric Mean (EBGM) from Multi‐Item Gamma Poisson Shrinker (MGPS) and proportional reporting ratio with Chi‐square (PRR, χ^2^) were used to calculate the disproportionality.

**Results:**

We filtered the query for indication and identified 80,316 reports with heart failure indication in FAERS during the analytical period. Among all the reports, sacubitril/valsartan was listed as primary suspected or secondary suspected drug in 29,269 cases. No significantly elevated reporting rates of narrow dementia were evident with sacubitril/valsartan. The EBGM05 for narrow dementia‐related AEs associated with sacubitril/valsartan was 0.88 and the PRR (χ^2^) was 1.22 (2.40). Similarly, broad demented complications were not over‐reported in the heart failure patients administrated with sacubitril/valsartan (EBGM05 1.11; PRR 1.31, χ^2^ 109.36).

**Conclusion:**

The number of dementia‐related cases reported to FAERS generate no safety signal attributable to sacubitril/valsartan in patients with heart failure for now. Further follow‐ups are still warranted to address this question.

## Graphical abstract

What is already known about this subject: There is an ongoing concern about the effect of sacubitril/valsartan on cognition, especially in case of long‐term administration. So far, results of related researches were still insufficient to show compelling evidence for long‐term cognitive safety of sacubitril/valsartan. What this study adds: This study mined the FAERS between 2015Q3 and 2022Q4 to analyze the association between sacubitril/valsartan and adverse events (AEs) involving dementia. Dementia‐related AEs were not over‐reported in the heart failure patients administrated with sacubitril/valsartan, suggesting that there is no safety signal generated attributable to sacubitril/valsartan for now.

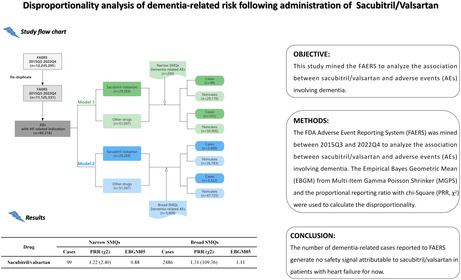

## 1 INTRODUCTION

Sacubitril/valsartan, the first‐in‐class angiotensin receptor‐neprilysin inhibitor (ARNI), was proven successful in treating patients with heart failure.^1^, ^2^ It was approved by the United States Food and Drug Administration (FDA) in 2015 with an indication of heart failure.^3^ Sacubitril could inhibit neprilysin and lead to decreased breakdown of vasoactive peptides with favorable actions for patients with heart failure.^4^, ^5^ However, neprilysin has other substrates in other systems including amyloid‐β peptides.^6^, ^7^ Inhibiting neprilysin could therefore lead to accumulation of certain amyloid‐β, a major pathological feature of Alzheimer's disease (AD), and might increase the risk of dementia‐related symptoms.^8^, ^9^ So far, results of related researches were still insufficient to show compelling evidence for long‐term cognitive safety of sacubitril/valsartan.^10^ We have, therefore, conducted a pharmacovigilance analysis for sacubitril/valsartan and adverse events (AEs) involving cognitive impairment using the real‐world FDA Adverse Event Reporting System (FAERS) database to update the long‐term cognitive safety profile of sacubitril/valsartan.

## 2 METHODS

### 2.1 Study design and data source

A retrospective, observational, pharmacovigilance study was performed on the de‐identified publicly available FAERS data. FAERS is a spontaneous database administered by the FDA and gathers information on AE reports that originate from different sources, including health‐care providers, patients, drug manufacturers, and others.^11^ Symptoms of AEs are coded using the Medical Dictionary for Regulatory Activities (MedDRA), an internationally standardized, clinically validated terminology.^12^ FAERS can be used to analyze unexpected patterns of AEs which are unlikely to be detected in clinical trials due to the limited number of participants.^11^, ^13^, ^14^

### 2.2 Data queries

All reports reported to the FAERS database between July 1, 2015 and December 31, 2022 were downloaded and accessed. We managed the raw FAERS data in local by Microsoft Access software (version 2021). Deduplication was applied prior to conducting any analysis. Only reports with indication of heart failure were included in this study. Each report was classified based on the following binomial factors: (1) “with” or “without” exposure to the administration of sacubitril/valsartan, which was classified as “primary suspected” or “secondary suspected” drug. (2) “With” or “without” the development of an AE category of interest, which was defined by using Standardized MedDRA Queries (SMQs) with “narrow” or “broad” preferred terms (PTs) related to dementia‐like AEs in the MedDRA 25.0. The precise terms used are detailed in Appendix 1.

### 2.3 Data analysis

**FIGURE 1:**
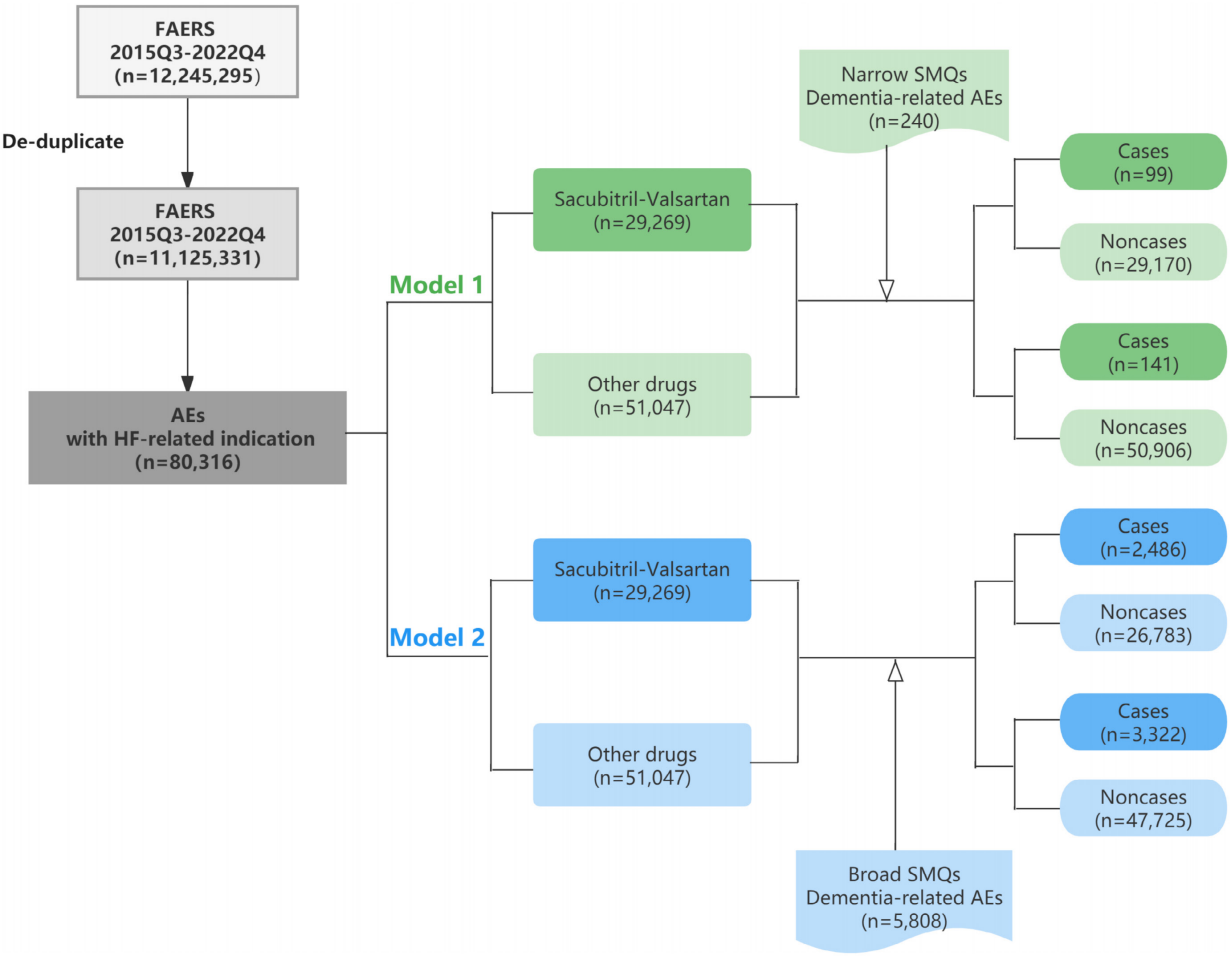
Flowchart of identifying cases and non‐cases from FAERS database.

A population‐based pharmacovigilance study using a case/non‐case approach was applied to analyze the risk of dementia for sacubitril/valsartan. This approach is a common system used in pharmacovigilance studies to identify drugs safety signals.^11^, ^12^, ^14^, ^15^ Mathematically, the idea of the case/non‐case system is to compare the frequency of an AE of interest in patients exposed to a specific drug (cases) with the reports of the same AE in patients who were not exposed to this drug (non‐cases).^16^, ^17^ This so‐called case/non‐case system can be considered a case–control study, and results can be measured by Multi‐Item Gamma Poisson Shrinker (MGPS) algorithm and the proportional reporting ratio (PRR) with its Chi‐square (χ^2^). Figure 1 shows the flow diagram for identifying cases and non‐cases from the FAERS database.

In our study, disproportionality was analyzed by calculating the Empirical Bayes Geometric Mean (EBGM) from MGPS and PRR (χ^2^). EBGM = a (a + b + c + d) / (a + c) / (a + b) in which a is the number of reports of dementia‐related AE for sacubitril/valsartan, b represents the reports for sacubitril/valsartan without reporting dementia, c is the number of the reports of dementia for all other drugs, d represents the number of the reports for all other drugs without reporting dementia. Signal was defined when the EBGM05 metric, a lower one‐sided 95% confidence limit of the EBGM ≥2.0.^17^ PRR = (a/[a + b])/(c/[c + d]). Signal was defined by the criterion of the PRR >2 with a χ^2^ > 4.^17^

### 2.4 Statistical analysis

Kolmogorov–Smirnov test was performed to evaluate the normality. Data are presented as mean ± standard deviation for normally distributed data and median (interquartile range [IQR]) for nonnormally distributed data. Frequencies and percentages are reported for categorical variables. Statistical analyses were performed using SPSS (version 28.0).

## 3 RESULTS

### 3.1 Data overview

Over the study period, a total of 12,245,295 AEs were available on FAERS database. Of these, 1,119,964 reports were excluded based on the exclusion criteria. We then filtered the query for indication with heart failure and identified 80,316 reports in FAERS during the analytical period, Overall, 29,269 AEs were found to be related to sacubitril/valsartan. Two hundred and forty reports related to narrow demented AEs and 5808 reports with broad demented AEs were documented. Among them, the sacubitril/valsartan was identified as the suspected drug causing narrow dementia in 99 reports and broad demented AEs in 2486 cases.

### 3.2 General characteristics

The demographic characteristics of the sacubitril/valsartan‐associated demented reports are presented in Table 1. Male reports were more frequent in all cases. In reports in which age was documented, the median age for reports with narrow SMQs is 76 (IQR 67–84) years, and 66 (IQR 58–76) years for broad cases. In most of the cases, weight was unknown or not reported. For reporter sources, health‐care providers reported 50.51% of the narrow cases, while consumer reported 70.43% of the broad cases. Most of the cases are reported after 2020.

**TABLE 1:** Clinical characteristics of reports with sacubitril/valsartan‐associated dementia.

Characteristics	Reports *n* (%)	
	Narrow SMQs	Broad SMQs
All reports	99	2486
Gender		
Female	43 (43.43)	1014 (40.79)
Male	48 (48.48)	1439 (57.88)
Unknown or missing	8 (8.08)	33 (1.33)
Age (year)		
< 18	0 (0.00)	1 (0.04)
18 ≤ and < 65	3 (3.03)	578 (23.25)
65 ≤ and < 75	14 (14.14)	366 (14.72)
≥ 75	18 (18.18)	357 (14.36)
Unknown or missing	64 (64.65)	1184 (47.63)
Median (IQR)	76 (67–84)	66 (58–76)
Weight (kg)		
< 50	8 (8.08)	26 (1.05)
50 ≤ and < 100	14 (14.14)	291 (11.71)
≥ 100	1 (1.01)	86 (3.46)
Unknown or missing	76 (76.77)	2083 (83.79)
Median (IQR)	64 (42–81.2)	79.4 (64–94.3)
Reporter		
Health‐care professional		
Physician	25 (25.25)	429 (17.26)
Pharmacist	10 (10.10)	19 (0.76)
Other	15 (15.15)	283 (11.38)
Non‐health‐care professional		
Consumer	49 (49.49)	1751 (70.43)
Unknown or missing	0 (0.00)	4 (0.16)
Reported year		
2015	0 (0.00)	10 (0.40)
2016	4 (4.04)	122 (4.91)
2017	6 (6.06)	169 (6.80)
2018	11 (11.11)	269 (10.82)
2019	16 (16.16)	309 (12.43)
2020	25 (25.25)	527 (21.20)
2021	16 (16.16)	514 (20.68)
2022	21 (21.21)	566 (22.77)
Onset time (d)		
Median (IQR)	136.5 (61.5–365)	87 (29–299.5)

### 3.3 Disproportionality analysis

**FIGURE 2:**
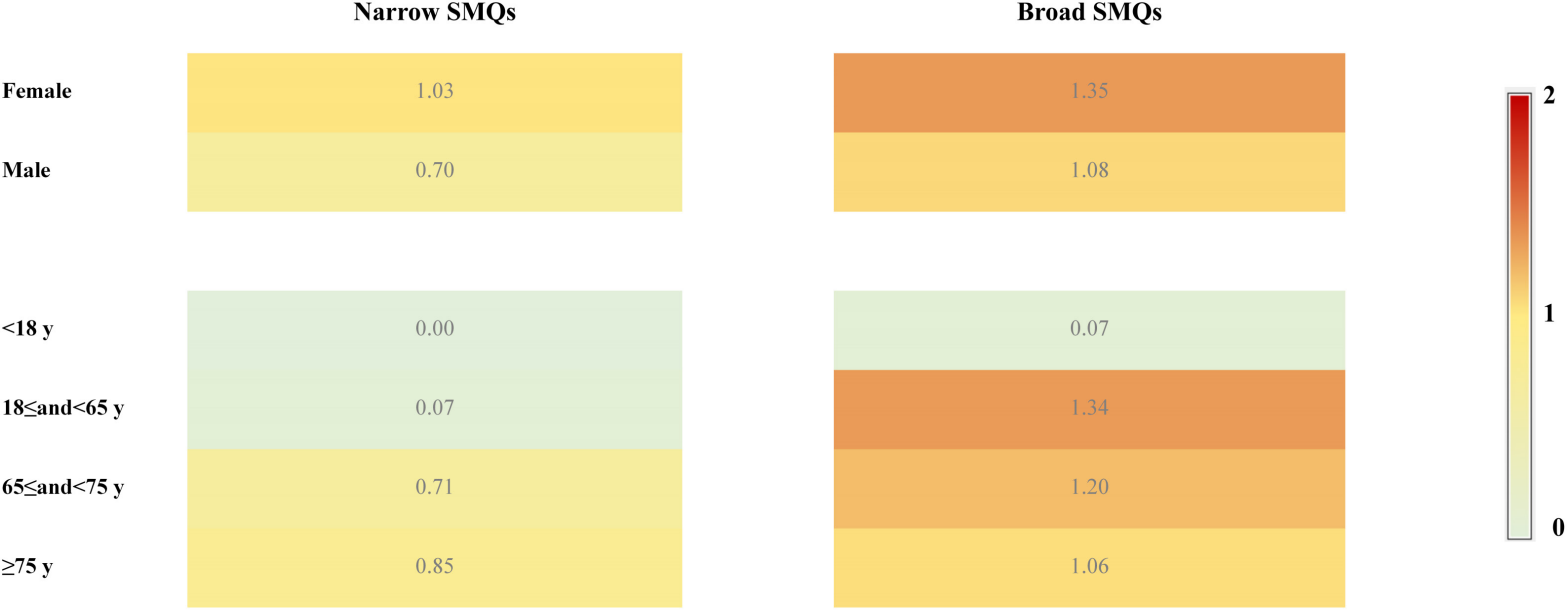
Subgroup disproportionality analysis of dementia following sacubitril/valsartan compared to all other drugs within heart failure patients from FAERS by age and sex (heatmap of EBGM05).

The results of disproportionality analysis are summarized in Table 2. The EBGM05 was 0.88 and the PRR (χ^2^) was 1.22 (2.40) for narrow dementia‐related AEs associated with sacubitril/valsartan, demonstrating that no over‐reporting of narrow dementia‐related AEs was identified in sacubitril/valsartan within heart failure patients. Similarly, broad demented complications were not over‐reported in the heart failure patients administrated with sacubitril/valsartan (EBGM05, 1.11; PRR 1.31, χ^2^ 109.36). To further access the individual characteristics, separate sub‐analyses based on age and sex were conducted too (Figure 2). No risk for narrow or broad dementia‐related AEs was noted in both females and males based on the results of EBGM05. Among reports in which age was documented, the EBGM05s for dementia (narrow and broad) were less than 2 in all specified age subgroups.

**TABLE 2:** Results of overall disproportionality analysis.

Drug	Narrow SMQs			Broad SMQs		
	Cases	PRR (χ2)	EBGM05	Cases	PRR (χ2)	EBGM05
Sacubitril/valsartan	99	1.22 (2.40)	0.88	2486	1.31 (109.36)	1.11

### 3.4 Time to onset

**FIGURE 3:**
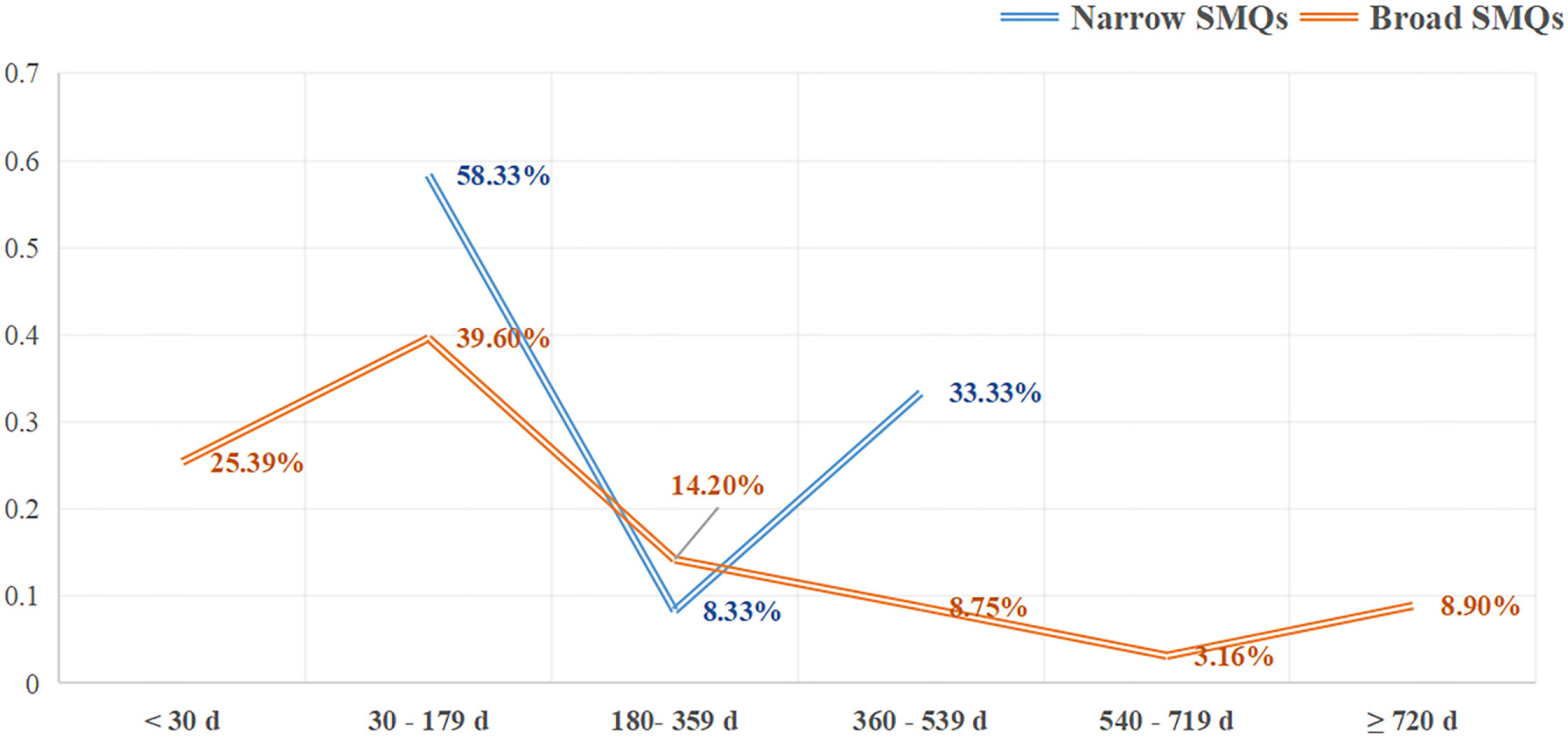
Time to onset of sacubitril/valsartan‐related demented AEs.

Onset time was analyzed using reports in which both drug_start_time and event_time were documented. Generally, the median adverse event onset interval for dementia was 136.5 (IQR 61.5–365) days for reports with narrow SMQs, and 87 (IQR 29–299.5) days for broad cases. The times to onset are summarized in Figure 3. In addition, most of the AEs of both narrow and broad dementia occurred in 30–179 days after taking sacubitril/valsartan.

## 4 DISCUSSION

There is an ongoing concern about the risk of cognition impairment for the sacubitril/valsartan, an angiotensin–neprilysin inhibitor, especially under long‐term administration.^5^, ^10^ Theoretical mechanisms of sacubitril/valsartan‐induced dementia have been proposed. Amyloid‐β plaque deposition is one of the most noticeable characteristics of AD. Neprilysin is a significant neuropeptidase and amyloid‐degrading enzyme.^18^ Inhibition of neprilysin by sacubitril might lead to accumulation of amyloid‐β in the central nervous system which is the pathognomonic feature of Alzheimer's type dementia.^6^, ^7^, ^18^ Some reports indicated that sacubitril can also inhibit the degradation of bradykinin.^19^ Numerous studies showed that bradykinin is also involved in the progress of AD and elevated bradykinin in plasma levels has been found in AD patients.^20^ There were numerous preclinical studies evaluating the theoretical risk of amyloid‐β accumulation following neprilysin inhibition. In two studies conducted by Langenickel et al.^7^ and Schoenfeld et al.^21^ the maximum concentrations of the active metabolite of sacubitril, sacubitrilat, measured in human cerebrospinal fluids (CSFs) and plasma were 58.8 and 19,600 ng/mL respectively, while in monkeys, maximum concentrations were 19.8 ng/mL in CSF and 34,400 ng/mL in plasma. Despite the concentrations of sacubitrilat in CSF are significantly lower than in plasma, some researchers still indicated that these values might adequate for effective inhibition of neprilysin in the brain since the IC50 of sacubitrilat for neprilysin inhibition is quite low.^10^ To be more specific, Schoenfeld et al.^21^ also conducted histopathological examinations to prove the effect of sacubitrilat on accumulation of amyloid plaques in different areas of the brain with a conclusion that no significant pathological alterations were noticed in the brain tissue.

Cannon and colleagues^22^ examined dementia‐related AEs in the Prospective comparison of ARNi with ACEi to Determine Impact on Global Mortality and morbidity in Heart Failure (PARADIGM‐HF) trial. In this study, 8399 patients aged 18–96 years were randomized and followed for a median of 2.25 years. The narrow SMQ search identified 27 dementia‐related AEs: 15 (0.36%) on enalapril and 12 (0.29%) on sacubitril/valsartan [hazard ratio (HR) 0.73, 95% confidence interval (CI) 0.33–1.59]. The broad search identified 97 (2.30%) and 104 (2.48%) AEs (HR 1.01, 95% CI 0.75–1.37), respectively. The short monitoring time and targeted populations might have significant influence over the negative result. A previous pharmacovigilance study^23^ have analyzed the association between sacubitril/valsartan and dementia‐related AEs with a conclusion that sacubitril–valsartan was not associated with a disproportionately high rate of short‐term dementia‐related adverse effect reports. To be noted, this published study only analyzed AEs submitted to the FAERS between July 2015 and March 2017. This observation time might be too short to monitor the risk since the average timeline for the development of AD‐related dementia spans over years to decades.^24^ Long‐term analysis assessing cognitive risks are required. Our study investigated the association between sacubitril/valsartan and dementia‐related AEs using 7‐year pharmacovigilance data from FAERS in real‐world setting.

There is a known suggestion that a substantial proportion of heart failure patients have concomitant cognitive problems.^25^ Epidemiological studies showed that the incidence of dementia in patients with heart failure is higher compared with the general populations.^26^, ^27^, ^28^, ^29^ Therefore, to be more specific, we filtered the query for an expanded heart failure indication in our study. The overall EBGM05s for narrow and broad dementia‐related AEs associated with sacubitril/valsartan were 0.88 and 1.11, respectively, demonstrating that no over‐reporting of dementia‐related AEs was identified in sacubitril/valsartan within heart failure patients. Studies have identified the epidemiology characteristics of dementia which is increasingly prevalent with advancing age and with little sex difference.^30^ Subgroup disproportionality analysis based on sex and age in our study detected no risk of dementia for sacubitril–valsartan in both males and females, and the risk did not exist in elderly people aged over 65 years and all other age groups.

Study limitations should be acknowledged. These limitations are mainly inherent to the nature characteristics of self‐reporting database. First of all, in most reports there is no demonstration of a causal relationship between the reported AE and drug exposure. The inability to make causal conformation is a limitation of all pharmacovigilance studies and cohort observational studies.^31^ Second, cases in FAERS might contain inaccurate and incomplete information such as age, weight, and onset time. For example, event onset time was missing in nearly half of the targeted cases in our study. Even we managed to access the onset time using reports in which both drug_start_time and event_time were documented, the incompleteness might have an impact on the preciseness of the analysis. Notwithstanding these limitations, disproportionality analysis still represents an invaluable method for identifying novel rare signals and monitoring drug safety. Many initial warnings about drug safety are primed by a disproportionality finding in the FAERS.^32^

In summary, our data indicated that in all reports from FAERS, no over‐reporting of dementia‐related AEs was identified in sacubitril/valsartan within heart failure patients. The number of dementia cases reported to FAERS did not generate a safety signal attributable to sacubitril/valsartan for now. Further long‐term studies are still warranted to follow up this question.

## AUTHOR CONTRIBUTIONS

Congqin Chen contributed to data analysis, interpretation, and writing. Lingqing Ding contributed to data analysis and revising. Fang Fu revised the manuscript. Jie Xiao conceived and designed this study.

## FUNDING INFORMATION

This work was partially supported by The Medical and Health Guidance Project of Xiamen (3502Z20214ZD1168).

## CONFLICT OF INTEREST STATEMENT

The authors declare that the research was conducted in the absence of any commercial or financial relationships that could be construed as a potential conflict of interest.

## Data Availability

The data supporting the conclusion of this article will be made available from the corresponding authors upon on reasonable request.

## Appendix 1

### Dementia‐like and related events

In this study, we systematically searched AE reports coded using the 25.0 version of the MedDRA using SMQs with “narrow” and “broad” preferred terms (PTs) related to dementia.

The narrow PTs used were: “behavioural and psychiatric symptoms of dementia”, “creutzfeldt‐jakob disease”, “dementia”, “dementia of the alzheimer's type, uncomplicated”, “dementia of the alzheimer's type, with delirium”, “dementia of the alzheimer's type, with delusions”, “dementia with lewy bodies”, “frontotemporal dementia”, “hippocampal sclerosis”, “korsakoff's syndrome”, “presenile dementia”, “progressive supranuclear palsy”, “senile dementia”, “variant creutzfeldt‐jakob disease”, “vascular dementia”.

The broad PTs used were: “abnormal behavior”, “abulia”, “activities of daily living impaired”, “affect lability”, “aggression”, “feeling abnormal”, “agitation”, “agnosia”, “amnesia”, “amnestic disorder”, “anterograde amnesia”, “learning disorder”, “apathy”, “hostility”, “aphasia”, “apraxia”, “borderline mental impairment”, “cerebral atrophy”, “symbolic dysfunction”, “cerebral atrophy congenital”, “change in sustained attention”, “cognitive disorder”, “confusional state”, “delirium”, “morose”, “delusion”, “delusional disorder, jealous type”, “delusional disorder, unspecified type”, “disinhibition”, “memory impairment”, “disorientation”, “disturbance in social behaviour”, “executive dysfunction”, “flat affect”, “hallucination”, “hypomania”, “illusion”, “impaired reasoning”, “inappropriate affect”, “initial insomnia”, “intelligence test abnormal”, “irritability postvaccinal”, “judgment impaired”, “learning disability”, “mental status changes”, “mood altered”, “mood swings”, “negativism”, “neuropsychological test abnormal”, “sexually inappropriate behaviour”, “personality change”, “prodromal alzheimer's disease”, “psychotic behavior”, “psychotic disorder”, “restlessness”, “social avoidant behaviour”, “somnambulism”, “somnolence”, “sopor”, “speech disorder”, “suspiciousness”, “thinking abnormal”, “transient global amnesia”, “vascular cognitive impairment”.

## References

[1] Ksiazczyk M , Lelonek M . Angiotensin receptor/neprilysin inhibitor‐a breakthrough in chronic heart failure therapy: summary of subanalysis on PARADIGM‐HF trial findings. Heart Fail Rev. 2020;25(3):393‐402. 31713710 10.1007/s10741-019-09879-x PMC7181555

[2] McMurray JJ , Packer M , Desai AS , et al. Dual angiotensin receptor and neprilysin inhibition as an alternative to angiotensin‐converting enzyme inhibition in patients with chronic systolic heart failure: rationale for and design of the prospective comparison of ARNI with ACEI to determine impact on global mortality and morbidity in heart failure trial (PARADIGM‐HF). Eur J Heart Fail. 2013;15(9):1062‐1073. 23563576 10.1093/eurjhf/hft052 PMC3746839

[3] Sauer AJ , Cole R , Jensen BC , et al. Practical guidance on the use of sacubitril/valsartan for heart failure. Heart Fail Rev. 2019;24(2):167‐176. 30565021 10.1007/s10741-018-9757-1 PMC6394573

[4] Vaduganathan M , Claggett BL , Desai AS , et al. Prior heart failure hospitalization, clinical outcomes, and response to Sacubitril/valsartan compared with valsartan in HFpEF. J Am Coll Cardiol. 2020;75(3):245‐254. 31726194 10.1016/j.jacc.2019.11.003 PMC7983315

[5] Wooster J , Cook EA , Shipman D . Psychiatric manifestations with Sacubitril/valsartan: a case report. J Pharm Pract. 2020;33(4):553‐557. 30991886 10.1177/0897190019842700

[6] Nalivaeva NN , Belyaev ND , Kerridge C , Turner AJ . Amyloid‐clearing proteins and their epigenetic regulation as a therapeutic target in Alzheimer's disease. Front Aging Neurosci. 2014;6:235. 25278875 10.3389/fnagi.2014.00235 PMC4166351

[7] Langenickel TH , Tsubouchi C , Ayalasomayajula S , et al. The effect of LCZ696 (sacubitril/valsartan) on amyloid‐beta concentrations in cerebrospinal fluid in healthy subjects. Br J Clin Pharmacol. 2016;81(5):878‐890. 26663387 10.1111/bcp.12861 PMC4834603

[8] Baranello RJ , Bharani KL , Padmaraju V , et al. Amyloid‐beta protein clearance and degradation (ABCD) pathways and their role in Alzheimer's disease. Curr Alzheimer Res. 2015;12(1):32‐46. 25523424 10.2174/1567205012666141218140953 PMC4820400

[9] Galo J , Celli D , Colombo R . Effect of Sacubitril/valsartan on neurocognitive function: current status and future directions. Am J Cardiovasc Drugs. 2021;21(3):267‐270. 33063249 10.1007/s40256-020-00445-7 PMC7561468

[10] Poorgolizadeh E , Homayouni Moghadam F , Dormiani K , Rezaei N , Nasr‐Esfahani MH . Do neprilysin inhibitors walk the line? Heart ameliorative but brain threatening! Eur J Pharmacol. 2021;894:173851. 33422508 10.1016/j.ejphar.2021.173851

[11] Ahmad J , Thurlapati A , Thotamgari S , et al. Anti‐cancer drugs associated atrial fibrillation‐an analysis of real‐world pharmacovigilance data. Front Cardiovasc Med. 2022;9:739044. 35498039 10.3389/fcvm.2022.739044 PMC9051026

[12] Kumar A . The newly available FAERS public dashboard: implications for health care professionals. Hosp Pharm. 2019;54(2):75‐77. 30923396 10.1177/0018578718795271 PMC6431724

[13] Ding L , Chen C , Yang Y , Fang J , Cao L , Liu Y . Musculoskeletal adverse events associated with PCSK9 inhibitors: disproportionality analysis of the FDA adverse event reporting system. Cardiovasc Ther. 2022;2022:9866486. 35140810 10.1155/2022/9866486 PMC8808238

[14] Zhai Y , Ye X , Hu F , et al. Updated insights on cardiac and vascular risks of proton pump inhibitors: a real‐world pharmacovigilance study. Front Cardiovasc Med. 2022;9:767987. 35282344 10.3389/fcvm.2022.767987 PMC8913586

[15] Yu RJ , Krantz MS , Phillips EJ , Stone CA Jr . Emerging causes of drug‐induced anaphylaxis: a review of anaphylaxis‐associated reports in the FDA adverse event reporting system (FAERS). J Allergy Clin Immunol Pract. 2021;9(2):819‐829 e812. 32992044 10.1016/j.jaip.2020.09.021 PMC7870524

[16] Rothman KJ , Lanes S , Sacks ST . The reporting odds ratio and its advantages over the proportional reporting ratio. Pharmacoepidemiol Drug Saf. 2004;13(8):519‐523. 15317031 10.1002/pds.1001

[17] Sakaeda T , Tamon A , Kadoyama K , Okuno Y . Data mining of the public version of the FDA adverse event reporting system. Int J Med Sci. 2013;10(7):796‐803. 23794943 10.7150/ijms.6048 PMC3689877

[18] Nalivaeva NN , Zhuravin IA , Turner AJ . Neprilysin expression and functions in development, ageing and disease. Mech Ageing Dev. 2020;192:111363. 32987038 10.1016/j.mad.2020.111363 PMC7519013

[19] Campbell DJ . Long‐term neprilysin inhibition – implications for ARNIs. Nat Rev Cardiol. 2017;14(3):171‐186. 27974807 10.1038/nrcardio.2016.200

[20] Ji B , Wang Q , Xue Q , Li W , Li X , Wu Y . The dual role of Kinin/Kinin receptors system in Alzheimer's disease. Front Mol Neurosci. 2019;12:234. 31632239 10.3389/fnmol.2019.00234 PMC6779775

[21] Schoenfeld HA , West T , Verghese PB , et al. The effect of angiotensin receptor neprilysin inhibitor, sacubitril/valsartan, on central nervous system amyloid‐beta concentrations and clearance in the cynomolgus monkey. Toxicol Appl Pharmacol. 2017;323:53‐65. 28315356 10.1016/j.taap.2017.03.014

[22] Cannon JA , Shen L , Jhund PS , et al. Dementia‐related adverse events in PARADIGM‐HF and other trials in heart failure with reduced ejection fraction. Eur J Heart Fail. 2017;19(1):129‐137. 27868321 10.1002/ejhf.687 PMC5248626

[23] Perlman A , Hirsh Raccah B , Matok I , Muszkat M . Cognition‐ and dementia‐related adverse effects with Sacubitril‐valsartan: analysis of the FDA adverse event report system database. J Card Fail. 2018;24(8):533‐536. 29746915 10.1016/j.cardfail.2018.04.010

[24] Verlinden VJA , van der Geest JN , de Bruijn R , Hofman A , Koudstaal PJ , Ikram MA . Trajectories of decline in cognition and daily functioning in preclinical dementia. Alzheimers Dement. 2016;12(2):144‐153. 26362597 10.1016/j.jalz.2015.08.001

[25] Cannon JA , Moffitt P , Perez‐Moreno AC , et al. Cognitive impairment and heart failure: systematic review and meta‐analysis. J Card Fail. 2017;23(6):464‐475. 28433667 10.1016/j.cardfail.2017.04.007

[26] Chitnis AS , Aparasu RR , Chen H , Kunik ME , Schulz PE , Johnson ML . Use of angiotensin‐converting enzyme inhibitors, angiotensin receptor blockers, and risk of dementia in heart failure. Am J Alzheimers Dis Other Demen. 2016;31(5):395‐404. 26705381 10.1177/1533317515618799 PMC10852826

[27] Matthews FE , Stephan BC , Robinson L , et al. A two decade dementia incidence comparison from the Cognitive function and Ageing Studies I and II. Nat Commun. 2016;7:11398. 27092707 10.1038/ncomms11398 PMC4838896

[28] Satizabal C , Beiser AS , Seshadri S . Incidence of dementia over three decades in the Framingham heart study. N Engl J Med. 2016;375(1):93‐94. 10.1056/NEJMc1604823 PMC6374770 27406362

[29] Adelborg K , Horvath‐Puho E , Ording A , Pedersen L , Sorensen HT , Henderson VW . Heart failure and risk of dementia: a Danish nationwide population‐based cohort study. Eur J Heart Fail. 2017;19(2):253‐260. 27612177 10.1002/ejhf.631 PMC5522185

[30] Fitzpatrick AL , Kuller LH , Ives DG , et al. Incidence and prevalence of dementia in the cardiovascular health study. J Am Geriatr Soc. 2004;52(2):195‐204. 14728627 10.1111/j.1532-5415.2004.52058.x

[31] Fadini GP , Sarangdhar M , Avogaro A . Glucagon‐like peptide‐1 receptor agonists are not associated with retinal adverse events in the FDA adverse event reporting system. BMJ Open Diabetes Res Care. 2018;6(1):e000475. 10.1136/bmjdrc-2017-000475 PMC5808638 29449951

[32] Blau JE , Tella SH , Taylor SI , Rother KI . Ketoacidosis associated with SGLT2 inhibitor treatment: analysis of FAERS data. Diabetes Metab Res Rev. 2017;33(8):e2924. 10.1002/dmrr.2924 PMC5950709 28736981

